# The Low Prevalence of Overweight and Obesity in Czech Breastfed Infants and Young Children: An Anthropological Survey

**DOI:** 10.3390/ijerph16214198

**Published:** 2019-10-30

**Authors:** Jitka Riedlová, Markéta Paulová, Jana Vignerová, Marek Brabec, Petr Sedlak, Dagmar Schneidrová

**Affiliations:** 1 Department of Anatomy, Third Faculty of Medicine, Charles University, Ruská 87, 100 00 Prague 10, Czech Republic; jitka.riedlova@lf3.cuni.cz; 2 Department of Hygiene of Children and Adolescents, National Institute of Public Health, Šrobárova 48, 100 42 Prague 10, Czech Republic; mpaulova@volny.cz; 3 National Lactation Centre, Thomayer Hospital, Vídeňská 800, 140 59 Prague 4, Czech Republic; jana.vigner@email.cz; 4 Institute of Computer Science, Czech Academy of Sciences, Pod Vodárenskou věží 271/2, 182 00 Prague 8, Czech Republic; mbrabec@cs.cas.cz; 5 Division of Child Health Promotion, Department of Hygiene, Third Faculty of Medicine, Charles University, Ruská 87, 100 00 Prague 10, Czech Republic; petr.sedlak-uk-prf@seznam.cz

**Keywords:** prevalence, overweight, obesity, breastfed infants, growth charts, primary health care

## Abstract

The aim of this study is to evaluate the prevalence of overweight and obesity in a sample of children who were exclusively or predominantly breastfed for at least 6 months compared to Czech references that were constructed based on a representative sample of children, regardless of their mode of feeding. Between 2008 and 2011, a longitudinal study on the growth of breastfed infants was carried out in the Czech Republic. Forty-three GP pediatricians addressed parents at 18-month preventive examinations and collected data on the families’ socio-economic conditions and the infants’ feeding conditions. The children were measured (length, weight, and head circumference), and anthropometric measurements from 10 previous preventive examinations were obtained from the health records. Out of the collected 1775 questionnaires, 960 children were selected according to the criteria of the WHO Multicentre Growth Reference Study. For the purpose of this study, 799 children who were exclusively or predominantly breastfed for at least 6 months were selected. We found that the proportions of children who were classified as overweight (>90th percentile) or obese (>97th percentile) at 6, 12, and 18-month examinations were far below the proportions of the Czech references. An update of the Czech references and growth charts is highly recommended by GP pediatricians for the valid assessment of growth and nutritional status, including a screening of overweight and obesity in primary preventive health care.

## 1. Introduction

Breastfeeding may have longer-term health benefits for the child, such as reducing the risk of overweight and obesity in childhood and adolescence [1]. The World Health Organisation (WHO) recommends that infants be exclusively breastfed for the first 6 months of life to achieve optimal growth, development, and health. Thereafter, to meet their evolving nutritional requirements, infants should receive nutritionally adequate and safe complementary foods, while continuing to breastfeed for up to 2 years or beyond [2,3].

Primary pediatric health care (PPHC), the foundation for prevention and health promotion in children, is an avenue for breastfeeding promotion and support. In light of the recent obesity epidemic, primary care physicians for children (GP pediatricians) are in a unique position for obesity prevention, diagnosis, and treatment [4].

Growth charts of the basic body parameters are an important tool for assessing the growth and nutritional status of children. Growth charts are also useful for evaluating the sufficiency of exclusive breastfeeding in the first 6 months of life and can be used as a tool for pediatricians when recommending the addition of complementary food. However, this decision making can be problematic if the growth charts do not represent the physiological growth patterns of the breastfed child correctly. The growth pattern of an exclusively breastfed child differs substantially from the growth of a child fed predominantly with infant formula [5,6]. The growth of breastfed children is faster than that of formula-fed children until the third month of age in terms of length and weight gain. Afterwards, their growth velocity decelerates so that roughly after 6 months of age they have a lower weight on average compared to formula-fed children [6,7]. Therefore, in 2006, the WHO recommended to replace the references of the National Centre for Health Statistics (NCHS), constructed on the basis of monitoring predominantly formula-fed children [8] according to the WHO growth standards [9]. The WHO growth standards for children from 0 to 59 months are a result of an international Multicentre Growth Reference Study (MGRS) performed between 1997 and 2003 in six countries (Brazil, Ghana, India, Norway, Oman, and the USA). This study covered healthy exclusively or predominantly breastfed children (at least 4 months in age), growing in optimal socio-economic conditions [10,11]. The WHO growth standards reflect the physiological patterns of the growth of breastfed children, regardless of their nationality or ethnic origin.

The Czech Republic belongs to the 17% of countries worldwide [12] where growth charts (references) are constructed on the basis of nationwide child growth monitoring, irrespective of their mode of feeding in an early age [13]. The WHO growth standards were compared to the currently used Czech growth references after the WHO Regional Office for Europe invited European countries to implement WHO growth standards. Significant differences between Czech growth references and WHO growth standards were found [5]. Consequently, three institutions (the Third Faculty of Medicine of Charles University, National Institute of Public Health, and Society for Primary Pediatric Care) collaborated on a study on the growth of Czech breastfed children between 2008 and 2011. Significant differences were found between the growth of Czech breastfed children and the WHO growth standards [14]. Czech breastfed children had different growth dynamics during their first 6 months of life. Moreover, during the second half of the first year of life they had a lower weight-for-age than the Czech growth references. However, a comparison of the Czech breastfed children with the WHO standards showed even larger differences. Czech breastfed children were longer and their weight-for-length was lower than the WHO growth standard. In general, the growth charts for Czech breastfed children up to 12 months adhere to the Czech growth references better than to the WHO growth standards. The previous study of Vignerová et al. [15] also documented that the implementation of WHO growth standards in Czech pediatric practice would lead to a significant increase in the number of children classified as “wasting” (below the third percentile in weight-for-length), especially in infants up to 5 months, which might result in a further clinical assessment of the child. Based on these findings, the authors recommended to continue using the Czech growth references (1991) for weight-for-age, weight-for-length, and BMI-for-age [16], and the Czech growth references (2001) for length/height-for-age and head circumference-for-age, in Czech pediatric practice [17]. GP pediatricians were instructed to consider the physiological patterns of a breastfed child mentioned earlier when evaluating growth and nutritional status [18].

The aim of this paper is to evaluate the prevalence of overweight and obesity in a sample of children who were exclusively or predominantly breastfed for at least 6 months compared to the Czech references.

## 2. Materials and Methods

### 2.1. Recruitment and Data Collection

Between 2008 and 2011, a longitudinal study on the growth and nutritional status of breastfed infants was developed in the Czech Republic (CR) in collaboration with two professional associations of GP pediatricians. Table 1 describes the time schedule of the research.

Seventy GP pediatrician trainers were invited to engage in the study. Ultimately, forty-three pediatricians participated in the data collection. Parents were recruited at the obligatory 18 months preventive examination and had the rationale of the research explained to them. Around 80% of parents agreed to participate, signed an informed consent form, and an interview was undertaken to obtain information on their family’s socio-economic conditions, mother’s age, education, family status, smoking status, weight and height, breastfeeding duration (exclusive or predominant), and the age they were introduced to supplemental and complementary foods. Data were recorded in a questionnaire form. Children were measured (length, weight, and head circumference), and anthropometric measurements from all previous preventive examinations (at birth, 2–3 days after discharge from the maternity hospital, at the age of 2 and 6 weeks, and at 3, 4, 6, 8, 10, 12, and 18 months) were obtained from the infants’ health records. Safety measures were taken while processing the data in order to ensure confidentiality.

Data were collected between April 2009 and May 2010. In March 2009, pediatricians obtained written instructions on the methodology of the anthropometric measurement and the administration of a questionnaire (Supplementary Materials). A total of 1775 completed questionnaires with 19,554 records of measurements were collected from pediatricians. Consequently, 960 children (471 boys and 489 girls) with 10,727 anthropometric measurements were selected according to the criteria of the WHO MGRS [19]. The mother was a non-smoker with at least secondary education, and each child was born from a single physiological pregnancy with a minimum birth weight of 2500 g and was exclusively or predominantly breastfed for at least 4 months; 92.2% children included in the study were exclusively (and 7.8%, predominantly) breastfed for at least 4 months. At the age of 6 months, 3.8% children were not breastfed and, at 12 months, 64.8% of children were still partially breastfed. According to the WHO definition, exclusive breastfeeding means that the infant receives only breast milk. No other liquids or solids are given (not even water, with the exception of an oral rehydration solution), nor any drops/syrups of vitamins, minerals, or medicines. The predominantly breastfed child gets extra water or non-milk liquids only [2].

For the purpose of the presented study, we selected from a sample of 960 children who met the MGRS criteria: 83.2% children (799 children—388 boys and 411 girls), who were exclusively or predominantly breastfed for at least 6 months (hereafter breastfed for 6 months). At the 6 month examination, 695 children among the selected 799 children were measured. At the 12 month examination, 731 children were measured, and 615 children were measured at the 18 month examination. The children were divided into two groups: (1) breastfed for 6 months (540 at 6 months, 562 at 12 months, 482 at 18 months), and (2) breastfed for longer than 6 months (155 at 6 months, 169 at 12 months, and 133 at 18 months). Each preventive examination was undertaken over the span of a month. The child came to the 6-month examination between 5.51 and 6.49 months of age; to the 12-month examination at between 11.51 and 12.49 months of age, and to the 18-month examination between 17.51 and 18.49 months of age.

Body length was measured while lying in a body-measuring bed; weight was measured on a calibrated children’s scale (naked child), and head circumference was measured with a band meter according to the standard methodology of Martin and Saler [20] by trained medical staff.

### 2.2. Czech References

The data of the sixth Nationwide Anthropological Survey of Children and Youth 2001 (hereafter NAS 2001), which are reference data for the body length-for-age and head circumference-for-age, were used for the analysis of the curves. The NAS 2001 was a cross-sectional survey that comprised the data of 58, 691 children, out of whom 8168 children were under 1 year of age. In this survey, it was neither discerned how the children were fed during the first months after birth, nor what their smoking status was or what the socio-economic conditions of their mother were [17]. For the analysis of the weight-for-age and weight-for-length curves, the data of the fifth Nationwide Anthropological Survey of Children and Youth 1991 (NAS 1991) were used. The NAS 1991 transversal survey comprised 90,910 children from birth to 18 years, out of whom 8844 children were under 1 year of age [16]. The children were included with no regard to the mode of their feeding practices, maternal smoking status, or education or other socio-economic data. The birth weight of the children was at least 1500 g. For the comparison of the body parameters of the children from our sample with the Czech references, we used the GrowthCZ software with valid growth references for the Czech children population [21]. According to the Czech references, the criteria for overweight corresponds with the cutoff of the >90th percentile and the obesity cutoff corresponds to the >97th percentile of the weight-for-length.

### 2.3. Statistical Analysis

The data were processed with EpiData Entry and EpiData Statistica and properly checked both formally and anthropologically. Individual growth curves for all the anthropometrical parameters of all children were checked. Only 10 children were excluded from the analysis due to significant shortcomings (missing data from previous preventive examinations). The proportions of the children with overweight and obesity were compared with proportions of the overweight and obese children in the Czech references. A chí-square test with an MCMC simulation was used, and overweight and obesity were considered as conditional probabilities [22].

### 2.4. Ethical Considerations

The present research was approved by the Ethics Committee of the Third Faculty of Medicine of Charles University on 28 January 2008. The mother or father provided written informed consent with the participation of the child in the study before filling out the questionnaire.

## 3. Results

Table 2 and Table 3 present the mean values for the standard deviation score (SDS) of body length and weight and the mean values of length and weight obtained at preventive examinations at 6, 12, and 18 months in children who were exclusively or predominantly breastfed for 6 months or longer. Highly significant differences were found between the mean values in boys and girls (*p* < 0.0001). The mean values of the SDS of body length corresponded to the 51st (at 6 months), 54th (at 12 months), and 55th (at 18 months) percentile of Czech references for boys, and to the 56th, 54th, and 52nd percentile in girls at 6, 12, and 18 months.

Mean values for the SDS of body weight reached below the 50th percentile and corresponded to the 49th (at 6 months), 40th (at 12 months), and 45th (at 18 months) percentile of Czech references for boys and to the 49th, 38th, and 43rd percentile for girls at 6, 12, and 18 months. Similarly, the mean values of the SDS of the weight-for-length both in boys (43rd, 41st, and 43rd percentile) and girls (39th, 36th, and 44th percentile) reached below the 50th percentile at 6, 12, and 18 months. From a clinical perspective, all mean values corresponded with the range of the mean values (from the 25th to 75t percentile). The biggest differences in weight and weight-for-length were found at the 12-month examinations, with lower values found in our sample compared to the Czech references.

**Figure 1:**
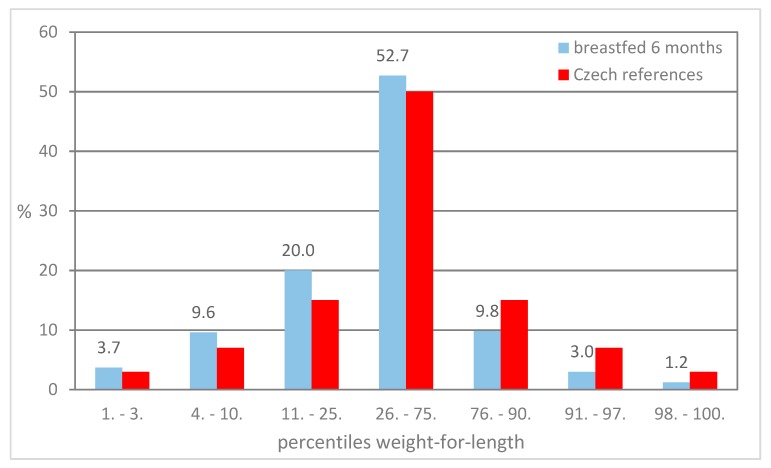
Proportion of children in the percentile categories of weight-for-length at 6 months.

**Figure 2:**
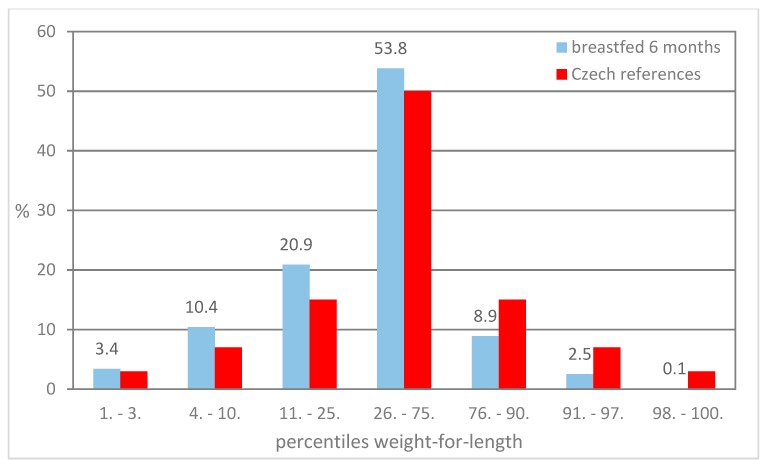
Proportion of children in the percentile categories of weight-for-length at 12 months.

**Figure 3:**
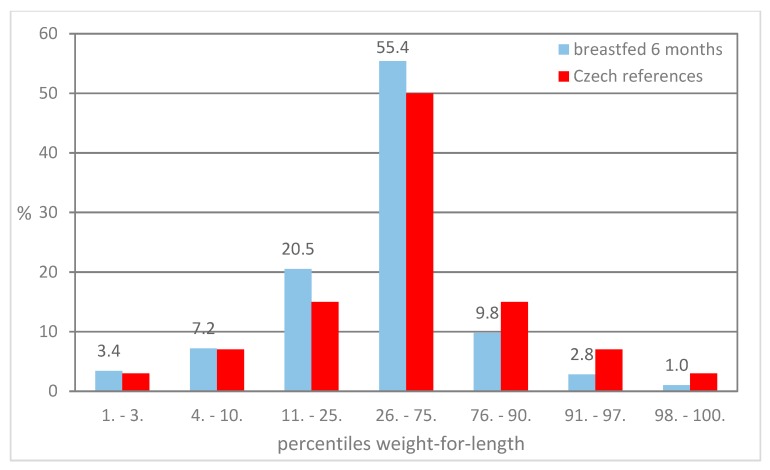
Proportion of children in the percentile categories of weight-for-length at 18 months.

Figure 1, Figure 2 and Figure 3 present the distribution of children breastfed for 6 months in particular percentile categories of weight-for-length during preventive examinations at the ages of 6, 12, and 18 months. The proportion of children classified below the 75th percentile is higher compared to the Czech references, and a lower proportion of children classified above the 75th percentile was found.

**Figure 4:**
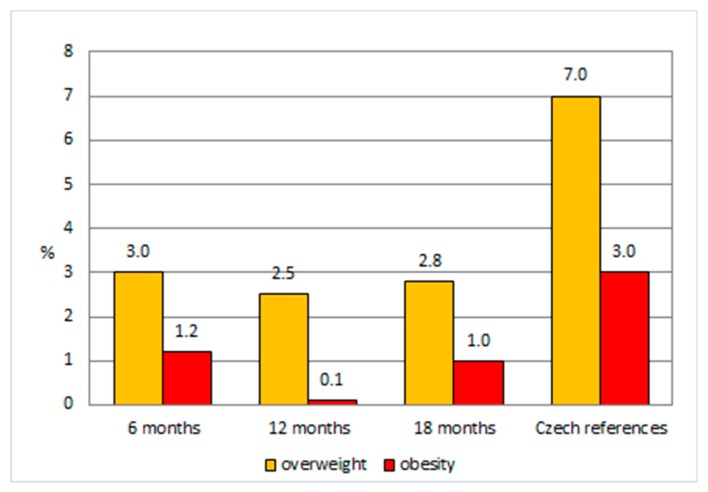
Prevalence of overweight and obesity in children breastfed for 6 months at 6, 12, and 18 months compared to the Czech references.

Figure 4 summarizes the findings in Figure 1, Figure 2 and Figure 3 and presents the prevalence of overweight and obesity in children breastfed for 6 months compared to the Czech references. Twenty-one children (3%) were classified as overweight (>90th percentile of weight-for-length) and eight children (1.2%) obese (>97th percentile of weight-for-length) at the 6-month examination. Eighteen children (2.5%) were overweight, and 1 boy was obese (0.1%) at the 12-month examination; and 17 children (2.8%) were overweight and 6 children obese (1%) at the 18-month examination. The proportions of children in our sample who were classified as overweight or obese were, at all three preventive examinations, far below the proportions of the Czech references: 7% were overweight (between the 91st and 97th percentile), and 3% were obese (between the 98th and 100th percentile).

Table 4 Compares the proportions of overweight and obese children in subgroups of children who were exclusively or predominantly breastfed for 6 months and longer than 6 months to the Czech references. No differences were found between the subgroups. However, in both subgroups, the proportions of overweight and obese children were lower than those in the Czech references. Only at the 12-month examination was the proportion of children with overweight and obesity significantly lower (*p* = 0.022) in children who were breastfed longer than 6 months compared to the Czech references.

Table 5 illustrates the development of the SDS of weight-for-length (related to Czech references) in individual children who were classified as overweight or obese. In our sample of 779 children who were breastfed for at least 6 months, only 12 children (1.5%) were classified as obese during at least one preventive examination at 6, 12, or 18 months. Among eight children classified as obese at 6 months, only three children were classified as overweight and one was child classified as obese at the 12 month examination; two children were classified as overweight and three children as obese at 18 months. Only one boy remained obese at all three examinations.

## 4. Discussion

In addition to providing all the nutrients an infant needs and protecting against common childhood diseases (diarrhoea and pneumonia), mounting evidence indicates that breastfeeding may have longer-term benefits, such as reducing the risk of overweight and obesity in childhood and adolescence [23].

Our study on a sample of children who were exclusively or predominantly breastfed for at least 6 months found that the proportions of children who were classified as overweight or obese at 6, 12, and 18 month preventive examinations were far below the proportions of the Czech references. The highest differences in weight and weight-for-length were found at 12 months examinations with lower values in our sample compared to the Czech references. Differences in the proportions of overweight and obese children in the subgroups of children who were exclusively and predominantly breastfed for 6 months and longer than 6 months were not significant. The smaller sample size in both subgroups may have reduced the statistical power. Moreover, the trend towards a lower response rate for the parents (around 80%) was observed during the data collection among GP pediatricians.

However, recent studies indicate that the more exclusively and the longer children are breastfed, the greater their protection from obesity. Rito et al. (2019) showed in the WHO European COSI study (round 4: 2015/2017) of 22 participating countries with nationally representative samples of 6 to 9 year-olds (*n* = 100,583) that, compared to children who were breastfed for at least 6 months, the odds of being obese were higher among children who were never breastfed or breastfed for a shorter period, both for general and exclusive breastfeeding [24]. Tambalis et al. (2018) found in a random sample of 5125 dyad children and their mothers, which was extracted from a national database in Greece, that exclusive breastfeeding for 6 months and longer (versus never) was associated with a lower risk of overweight in childhood (8 years old) [25]. In Greece, previous findings showed that children who were exclusively breastfed were 0.49 and 0.54 times less likely to be overweight at the ages of 6 and 12 months, compared to those who were not breastfed [26]. Wallby et al. (2017) studied a potential link between breastfeeding in infancy and obesity at age 4 in a sample of 30,508 infants born during 2002–2007 from the databases of the Preventive Child Health Services in two Swedish counties and from national registers. The authors concluded that a breastfeeding duration of at least 4 months may contribute independently to a reduced risk of childhood obesity at 4 years [27]. The results of the previous meta-analysis suggest that breastfeeding is a significant protective factor against obesity in children. The link between breastfeeding and childhood obesity was slightly different in studies that used different breastfeeding types. This may be because the original data from several studies did not distinguish between exclusive and partial breastfeeding, thereby reducing the precision of the analysis. In addition, a stepwise gradient of the decreasing risk of obesity by increasing duration of breastfeeding was observed, indicating a change to dose-response effect [28,29]. Weng et al. (2012) identified 30 prospective studies, with some evidence associating the early introduction of solid foods with childhood overweight. There is, however, conflicting evidence for the duration of breastfeeding [30].

Cardiovascular disease, type 2 diabetes, obesity-attributable cancers, osteoarthritis, and psychological disturbances generate much to the morbidity and years of life lost associated with increasing levels of obesity. There is a strong rationale for intervening during early life in infants at risk of developing childhood obesity, and, to date, interventions have focused on nutritional modification by supporting parents regarding, for example, healthy eating and breastfeeding. Both the Canadian Pediatric Society [31] and the American Academy of Pediatrics [32] advocate that all typically developing children aged 2 years and older have their growths monitored to screen for under-development, wasting, overweight, and obesity. However, in many countries, early life intervention is not routine clinical practice. Although in the USA the Institute of Medicine has introduced early childhood obesity prevention guidelines [33] suggesting that healthcare professionals (HCPs) should undertake regular growth monitoring and consider obesity risk factors during infancy, there is evidence in both the UK and the USA that HCPs are reluctant to diagnose obesity in infants [32].

In the Czech Republic, preventive examinations in the primary pediatric health care are mandatory, according to the directive of the Ministry of Health (No. 70/2012). Routine anthropometric measurements are undertaken 2–3 days after discharge from the maternity hospital, at the ages of 2 and 6 weeks and 3, 4, 6, 8, 10, 12 and 18 months, as well as every second year from the age of 3 to 18 years. Percentile charts used for the evaluation of growth and nutritional status are constructed based on the data of the Sixth Nationwide Anthropological Survey of Children and Youth (NAS 2001), which provide reference data for the body’s length-for-age and head circumference-for-age [17], as well as data for the fifth Nationwide Anthropological Survey of Children and Youth (NAS 1991), which offer reference data for weight-for-age and weight-for-length [16]. Since the children of the reference population were included in the nationwide anthropological surveys with no regard to the modes of their feeding practices, pediatricians are instructed to consider the specific development of growth in a breastfed child via the GrowthCZ software with valid growth references for the Czech child population [21]. Besides the regular monitoring of growth and nutritional status (including the early identification of overweight and obese children), GP pediatricians are expected to provide consistent information on breastfeeding and artificial feeding when necessary and introduce complementary foods after 6 months. Due to the lack of staff and time in primary health care, they can also refer mothers to the community network of lactation consultants (qualified nurses, midwives, physicians, or mothers) who are trained in the National Lactation Centre in Thomayer Hospital in Prague to provide counselling on breastfeeding and appropriate infant feeding practices.

Similarly, in Scotland, health visitors have been identified for their key roles in providing infant feeding advice and for their potential to encourage appropriate feeding practices for preventing obesity. A health visitor (HV) is a qualified nurse (or midwife) who has completed specialist training in children and family health. HVs offer support and advice regarding the wellbeing of children until their school years. HVs routinely discuss infant feeding with parents/caregivers as part of the universal Health Visiting Pathway in Scotland, from pre-birth to preschool. This program consists of 11 home visits to be delivered by HVs to all families, eight of which are within the first year of life, as well as three Child Health Reviews between 13 months and 4–5 years. Feeding and diet are routinely discussed with families as infants progress to solid foods. However, these infant nutrition conversations are not systematically delivered or recorded and could provide an opportunity to collect routine data related to nutrition and dietary diversity. Moreover, health workers’ dietary counselling has been shown to have a potentially positive influence on infant feeding practices. Moreover, establishing routine, consistent conversations around feeding practices could provide an opportunity for intervention and/or evaluations of earlier interventions. Within the majority of National Health Service (NHS) board areas across Scotland, HV teams now use electronic patient recording systems via tablets and computers. EMIS Web is the key cornerstone Electronic Patient Record Application in Community Services in NHS Greater Glasgow and Clyde (GGC), providing a single shared record for community-based children’s services. Electronic record keeping is advocated to improve administrative efficiency and time management, and this system provides a suitable opportunity to incorporate consistent and valid data collection. The widespread routine collection of similar information could facilitate the maintenance of nationally representative and up-to-date nutritional data on demand [34].

In the Czech Republic, we have considered, together with the Ministry of Health (the National Institute of Public Health and General Health Insurance Company), a way to establish a similar system for the electronic collection of anthropometric and infant feeding data in primary pediatric practice, which will facilitate an update of the Czech references (the eighth Nationwide Anthropological Survey 2021). The findings from our longitudinal survey of 2008–2011 strongly justify the need for an update of the Czech growth references [14,15,18].

## 5. Conclusions

In a sample of children who were exclusively or predominantly breastfed for at least 6 months, we found that the proportions of children who were classified as overweight or obese at 6, 12, and 18 months after preventive examinations were far below the proportions of the Czech references that were constructed based on a representative sample of children, regardless of their mode of feeding. The biggest differences in weight and weight-for-length were found at the 12 month examinations. The proposed nationwide anthropological survey might provide more relevant findings based on a representative sample of the population and will enable the Czech references and growth charts to be updated, which is highly requested by GP pediatricians for the routine assessment of growth and nutritional status, including the screening of overweight and obesity.

## Figures and Tables

**Table 1:** Time schedule of the study.

Time Period	Activity
2008	Preparation of the study.
February–March 2009	GP pediatricians addressed by professional associations at several educational meetings.
March 2009	GP pediatricians who agreed to participate in the study got written instructions on the methodology of anthropometrical measurements and administration of the questionnaire.
April 2009–May 2010	Data collection by GP pediatricians.
May–June 2010	Collection of questionnaires from GP pediatricians.
June 2010	Data processed with the EpiData Entry.
July–August 2010	Formal and logical control of data; corrections based on consultations with GP pediatricians.
August–December 2010	The construction and control of the individual growth curves of all children; corrections based on consultations with GP pediatricians.
January–March 2011	Sampling of breastfed children, statistical analysis of data, and construction of growth curves of breastfed children.

**Table 2:** The mean body length and mean SDS of body length at 6, 12, and 18 months in children breastfed for 6 months.

Preventive Examination (months)	Sex	n	Body Length (cm)				SDS Body Length	
			Mean	SD	Min	Max	Mean	SD
6	boys	334	68.9	2.46	62.0	77.0	0.04	0.80
	girls	362	67.2	2.43	60.0	78.0	0.13	0.83
12	boys	355	77.1	2.60	70.0	86.0	0.08	0.82
	girls	376	75.4	2.65	67.0	83.0	0.06	0.85
18	boys	313	83.5	3.05	74.0	91.5	0.14	0.89
	girls	302	81.8	2.84	72.0	90.0	0.04	0.85

SDS = standard deviation score.

**Table 3:** The mean body weight and mean SDS of body weight at 6, 12, and 18 months for children breastfed for 6 months.

Preventive Examination (months)	Sex	n	Body Weight (g)				SDS Body Weight	
			Mean	SD	Min	Max	Mean	SD
6	boys	335	7926.3	895.49	5530	11,450	−0.08	0.94
	girls	367	7288.5	796.24	5290	10,070	−0.12	0.92
12	boys	355	10,000.6	1035.27	7600	13,950	−0.31	0.90
	girls	376	9283.8	963.38	6680	12,570	−0.39	0.93
18	boys	314	11,574.6	1241.60	8000	15,600	−0.18	0.95
	girls	302	10,877.2	1179.60	7480	15,440	−0.25	0.96

SDS = standard deviation score.

**Table 4:** Proportion of overweight and obese children breastfed for 6 months and longer than 6 months compared to the Czech references at 6, 12, and 18 months.

Preventive Examination (months)	Weight-for-Length (percentiles)	Excl.BF = 6 m		Excl.BF > 6 m		Czech References
		n	%	n	%	
6	overweight	18	3.3	3	1.9	7
	obesity	6	1.1	2	1.3	3
12	overweight	14	2.5	4	2.4	7
	obesity	1	0.2	0	0.0	3
18	overweight	17	3.5	0	0.0	7
	obesity	4	0.8	2	1.5	3

Excl.BF = 6 m: exclusive breastfeeding for 6 months. Excl.BF > 6 m: exclusive breastfeeding for longer than 6 months.

**Table 5:** Development of the SDS of the weight-for-length in children classified as overweight or obese at 6, 12, and 18 months.

Sex	SDS of Weight-for-Length			Breastfeeding (months)	
	6 Months	12 Months	18 Months	Exclusive or Predominant	Total
girl	1.10	0.40	2.04	6	10
boy	2.15	0.56	1.86	6	12
girl	−0.17 ^1^	0.69	2.16	6	11
girl	1.08	1.28	2.32	6	11
girl	1.29	0.76 ^2^	2.30	6	8.25
girl	2.03	1.54	1.35	6	14
girl	2.24	0.92	0.51	6	11
boy	2.88	1.56	1.20	6	18
boy	2.18	1.63 ^3^	1.23	6	8
boy	2.15	2.52	2.42 ^4^	6	18
girl	2.13	1.28	2.08	8	12
boy	2.21	1.49	2.08	8	18

Colors of boxes: light grey = overweight, dark grey = obese, SDS = standard deviation score. ^1^ 6 months examination at the age of 8.05 months. ^2^ 12 months examination at the age of 13.15 months. ^3^ 12 months examination at the age of 12.89 months. ^4^ 18 months examination at the age of 19.79 months.

## Supplementary Materials

The following are available online at https://www.mdpi.com/1660-4601/16/21/4198/s1. Questionnaire on Research on child growth in relation to infant feeding.
