# Supplementary material for: The Low Prevalence of Overweight and Obesity in Czech Breastfed Infants and Young Children: An Anthropological Survey
Source: Int J Environ Res Public Health. 2019 Oct 30;16(21):4198. doi: 10.3390/ijerph16214198 (PMC6862631; doi:10.3390/ijerph16214198)
Supplement: Supplementary file 1 [file ijerph-16-04198-s001.pdf]

Research on child growth in relation to infant feeding

Instructions for GP pediatricians: When filling in the questionnaire, please write the relevant data in the grey field or choose the proper answer and cross **only one answer**.

**Address of the facility** where the parent was contacted:<br><br>**Zip code:**	**No. of inhabitants in the residence of the child**	Up to 4,999		(1)
		5,000–49,999		(2)
		50,000 and more		(3)

Identification data of the child

**First name of the child**										
**Date of birth of the child**			**Sex**	boy		(1)	**Nationality of the child**	Czech		(1)
day:	month:	year:		girl		(2)		other		(2)

Data of the mother

1	Age of the mother			years
2	Body height of the mother			cm
3	Body weight of the mother			kg
4	Family status of the mother	married		(1)
		divorced		(2)
		single		(3)
5	Education (highest attained)	primary		(1)
		vocational		(2)
		secondary and postsecondary		(3)
		bachelor		(4)
		master		(5)
6	Smoking status of the mother	never smoked		(1)
		quitted before pregnancy		(2)
		quitted during pregnancy		(3)
		quitted after birth		(4)
		current smoker		(5)

Data on child birth

7	Birth order (of the child of the biological mother)		
8	Gestational age (at what week of pregnancy was the child born)		week
9	Birth weight of the child		g
10	Birth length of the child		cm

TURN

Feeding of the child

11	First suckling from the breast	0–2 hours after birth				(1)
		2–12 hours after birth				(2)
		12–24 hours after birth				(3)
		24–48 hours after birth				(4)
12	At the discharge from maternity hospital	exclusive breastfeeding				(1)
		breastfeeding with supplementation				(2)
		infant formula				(3)
13	**Total** duration of breastfeeding (in months and weeks)			months		weeks
14	When did you introduce complementary food to the child?	tea		months		weeks
		other liquids besides milk (water, juice, etc.)		months		weeks
		fruits		months		weeks
		vegetables		months		weeks
		meat		months		weeks
		cereals		months		weeks
		infant formula		months		weeks
15	Reason for supplementation	child not thriving				(1)
		„hungry child“				(2)
		illness of the child				(3)
		illnes of the mother				(4)
		medical indication				(5)
		appropriate age of the child				(6)
16	Vegetarianism of the mother		yes			(1)
			no			(2)
17	Vegetarianism of the child		yes			(1)
			no			(2)

Data from the Health and Vaccination Records of the Child based on measurements of the GP pediatrician (current and all previous preventive examinations, other measurements)

date	length (cm)	weight (g, kg)	head circumference (cm)	comments

I was instructed on the conditions of providing personal data for this research (voluntary participation, providing further information on demand). I agree with the measurements of the child and to provide relevant data for anonymous statistical analyses.

Date……………….. Signature (mother/father)……………………………
